# 3D Integrated Circuit Cooling with Microfluidics

**DOI:** 10.3390/mi9060287

**Published:** 2018-06-07

**Authors:** Shaoxi Wang, Yue Yin, Chenxia Hu, Pouya Rezai

**Affiliations:** 1 School of software and microelectronics, Northwestern Polytechnical University, Xi’an 710072, China; yinyue@nwpu.edu.cn; 2 Lassonde School of Engineering, York University, Toronto, ON M3J 1P3, Canada; pouya.rezai@lassonde.yorku.ca; 3 Research & Development Institute of Northwestern Polytechnical University, Shenzhen 518057, China; 4 School of Pharmaceutical Science, Guangzhou University of Chinese Medicine, Guangzhou 510006, China; 20010614@gzucm.edu.cn

**Keywords:** microfluidics, 3D, integrated circuits, cooling

## Abstract

Using microfluidic cooling to achieve thermal management of three-dimensional integrated circuits (ICs) is recognized as a promising method of extending Moore law progression in electronic components and systems. Since the U.S. Defense Advanced Research Projects Agency launched Intra/Inter Chip Enhanced Cooling thermal packaging program, the method of using microfluidic cooling in 3D ICs has been under continuous development. This paper presents an analysis of all publications available about the microfluidic cooling technologies used in 3D IC thermal management, and summarized these research works into six categories: cooling structure design, co-design issues, through silicon via (TSV) influence, specific chip applications, thermal models, and non-uniform heating and hotspots. The details of these research works are given, future works are suggested.

## 1. Introduction

With the feature size of integrated circuits (ICs) scaling down towards the extreme of traditional micro/nano fabrication, the 3D IC technique is a promising method of extending the Moore law [1]. Three-dimensional ICs can alleviate the interconnection bottleneck that currently exists at the nanometer scale and continue the law’s progression. This next-generation technology is promising for multiple cores, memory, and logic units that are stacked vertically and interconnected in a single unit to increase clocking speed, reducing transmission losses, power consumption, and footprint area [2].

**Figure 1:**
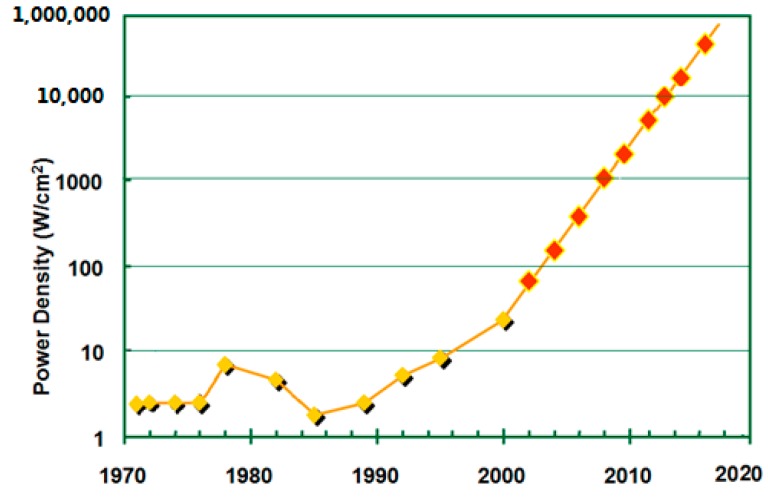
The power density trend for a single package.

However, 3D ICs also present many serious challenges that need to be overcome to make the technology useful. The greatest challenge is overheating because of an increasing power flux and a higher thermal resistance. This 3D IC technology may lead to the alienation of stacked-structure hotspots and the increase of local heat fluxes, and global chip temperature, hence degrading chip performance and reliability. The reliability, performance, and power dissipation of interconnects and transistors are heavily dependent on the operating temperature. Therefore, chip-level cooling with large power dissipation in high performance chips has become imperative. International Technology Roadmap for Semiconductors (ITRS) projects that the power density of a single package will increase to 10^6^ W/cm^2^ for performance applications in 2018 [3]. Such a trend is shown in Figure 1. Power density is increased greatly as more chips are integrated into a package.

Consequently, effective cooling structures used in 3D IC technologies need to be developed in order to benefit beyond the Moore law [4]. A number of chip cooling methods, which include passive and active cooling methods, have been proposed. Passive methods include thermal conduction (pastes, metal lines, and vias), natural convection (finned heat sinks and ventilation slots), radiation (coatings and paints), heat pipes, and thermosyphons [5]. These passive devices are easily designed and generally inexpensive to implement, but typically perform worse than active-cooling devices. Active cooling requires input power, which requires external components such as forced convection devices (fans and nozzles), pumped loops (heat exchangers and cold plates), and refrigerators (Peltier thermoelectric and vapor-compression-based) [5]. However, these cooling methods cannot be embedded in 3D IC structures with a small enough size or effective cooling, and are still unable to address the varying thermal profiles of an IC.

Microfluidics, also called lab-on-a-chip, is the science and technology of systems that process or manipulate small amounts of fluids, using channels or flat platforms with dimensions of tens to hundreds of micrometers [6]. In 2004, American Business 2.0 magazine named microfluidics as one of the seven new technologies that change everything, stating that such innovations were already here and were about to hatch some of tomorrow’s greatest business opportunities [7]. In July 2006, Nature magazine published a special issue for microfluidics, presenting the origins, the future, and the basic principle and applications of microfluidics, and recognized it as “the technologies of the century” [8]. Moreover, most research works focus on applications in biology, chemistry, materials science, and medical science.

More recently, microfluidics cooling has been demonstrated as promising for 3D ICs with high power density. Since 2012, the U.S. Defense Advanced Research Projects Agency (DARPA) has worked on an Intra/Inter Chip Enhanced Cooling (ICECool) thermal packaging program to solve cooling limitations and remove significant barriers so as to continue Moore law progression in electronic components and systems. The program adopts microfluidic cooling as aggressive thermal management techniques that directly cool the heat generation sites in the chip, substrate, and/or package [9]. With the promotion of the ICECool program, microfluidic technologies have been developed to cool 3D ICs in the last few years. There are 85 publications available concerning 3D ICs with microfluidics. Five dissertations and 80 journal or conference publications have been presented on basic principles and methods. These research works can be summarized into six categories: cooling structure design, co-design issues, through silicon via (TSV) influence, specific chip application, thermal models, and non-uniform heating and hotspots. Section 2 focuses on cooling structure design. Section 3 describes co-design issues in microfluidic cooling for 3D ICs. Section 4 shows how the design of TSVs in microfludic cooling can be optimized. Section 5 focuses on microfluidic cooling for specific chip applications. Section 6 presents thermal models, characteristics, and transmissions. Section 7 focuses on non-uniform heating and hotspots. The last section summarizes the review and concludes.

## 2. Microfluidic Cooling Structure and Manufacture

**Figure 2:**
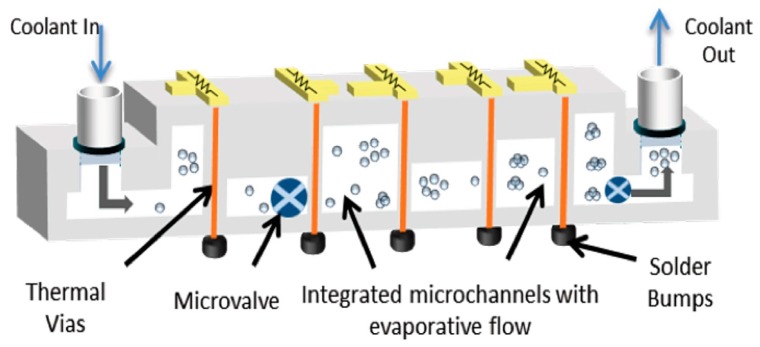
A cross-Sectional conceptual schematic of an ICECool device.

ICECool programs are working to create many micro/nano microfluidic channels and structures embedded in 3D ICs with high thermal conductivity, as well as thermoelectric materials to link on-chip hotspots to microfluidically cooled microchannels. The proposed intra/inter chip-enhanced cooling methods are required to be compatible with materials, fabrication procedures, and thermal management needs of homogeneous and heterogeneous integration in 3D chip stacks. A conceptual ICECool structure is given in Figure 2 [10,11].

**Figure 3:**
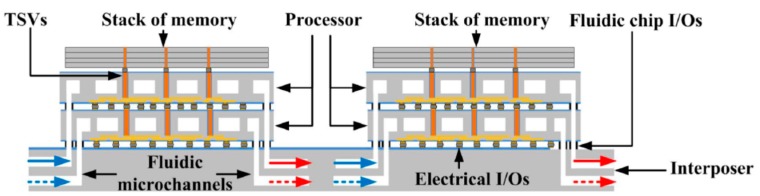
The tier-specific microfluidic cooling technology in 3D stacks. Reproduced with permission from [15].

Micropores and microchannels are directly fabricated into intra-chips [12]. The interchip approach uses the microgap as a cooling channel between chips in three-dimensional stacks [13,14]. Based on the conceptual model of the ICECool program, several different structures of microfluidic cooling embedded into 3D ICs have been presented. Yue Zhang et al. proposed tier-specific microfluidic cooling technology, which was experimentally demonstrated in a 3D stack, shown in Figure 3 [15]. The tier-specific cooling approach, compared with conventional microfluidic cooling, can reduce the pumping power by 37.5%, preventing overcooling, when an operating temperature is specified.

In Figure 3, the blue arrows indicate cold coolants and red arrows indicate hot coolants. To develop the structure in Figure 3, Minhaj Hassan et al. attempted to study the influence of temperature on circuit performance and the advantages of using microfluidics technology for continued performance scaling. Experimental results certified that conventional air cooling solutions limit 3D stacks [16]. To solve stack bonding, Yassir Madhour et al. presented a patterned die-to-die thin film bonding method for 3D chip stacks with integrated microfluidic cooling. The method was developed and successfully tested [17]. Paragkumar et al. then demonstrated the manufacture and characterization of a thick silicon interposer, which had low-loss polymer-embedded vias and two dices. The integrated microfluidic heat sink and fluidic and electrical I/Os were embedded into a silicon interposer for high-performance 3D system integration [18].

**Figure 4:**
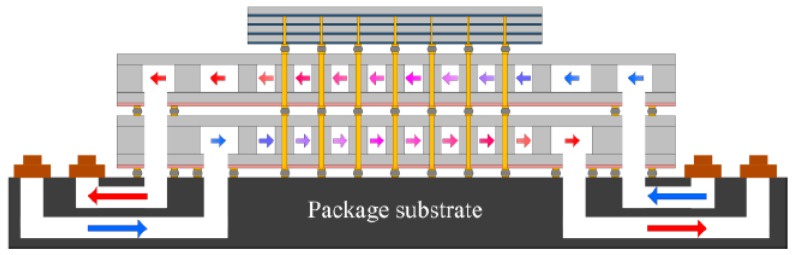
A 3D system with independent microfluidic cooling. Reproduced with permission from [21].

Modified microfluidic cooling structures, shown in Figure 4, have also been studied. Each chip had its own fluidic inlet and outlet. Flow direction and flow rate were modified independently for each die based on individual demands [19,20,21]. This approach achieved independent cooling for each tier and can be used for different temperature distributions and velocities.

**Figure 5:**
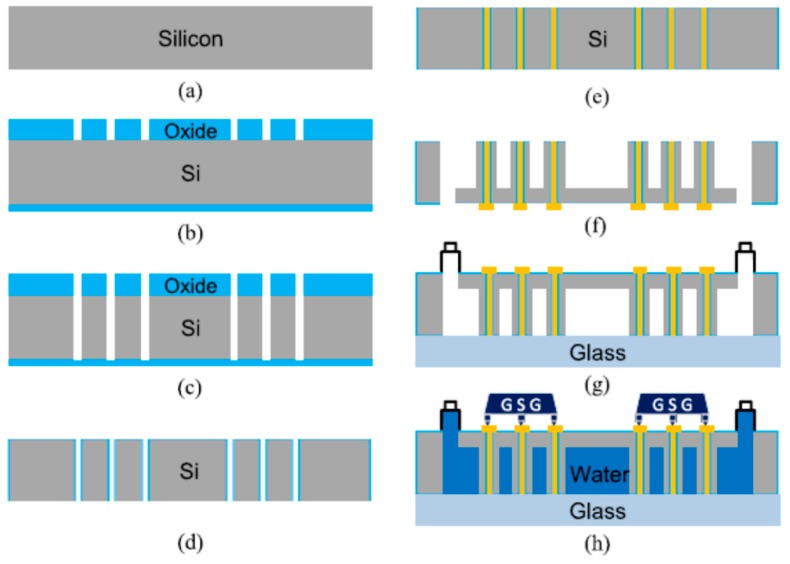
Schematic of the overall fabrication process.

Through silicon vias (TSVs) embedded in the 3D ICs require more complicated processes for microfabrication. Typical processes are shown in Figure 5. The processes started with the deposition of silicon dioxide followed by metal depositing. A thin chrome layer was chosen as an etch mask, as shown in Figure 5b. Using the chrome mask, the silicon dioxide layer was etched, and using a CR-7S chrome etchant, the remaining chrome was removed. Figure 5c shows the resulting silicon dioxide as an etch mask. The high-aspect-ratio Bosch process etched via holes through the silicon wafer. Figure 5d shows that the wet oxidation isolates the vias from the silicon substrate. Titanium and copper seed layers were deposited using an e-beam evaporator at the backside of the wafer. Moreover, chemical mechanical polishing will need to be removed the overburden (Figure 5e). A micropin-fin heat sink structure, shown in Figure 5f, will then be achieved. Finally, a glass slide is assembled into the testbed with fluidic inlet/outlets for fluid delivery, shown in Figure 5g [21].

## 3. Co-Design of Microfluidic Cooling in 3D Integrated Circuits (ICs)

A new strategy integrates the computational, electrical, physical, thermal, and reliability aspects of a system. The unification of these diverse aspects of ICs is called co-design. The independent optimization and design of each aspect leads to sub-optimal designs considering the lack of understanding of cross-domain interactions and their impacts on the feasibility region of the architectural design space. Thus co-design enables optimization with efficient design and high-performance configurations.

Although the co-design strategy is becoming increasingly imperative in IC design, 3D ICs with efficient microfluidic cooling has become even more critical. The interlayer coupling with cooling structures, and a higher degree of connectivity between components, exacerbates the interdependence between physical design parameters, architectural parameters, and a multitude of metrics of interest such as performance, hotspot distribution, power, and reliability. The embedded microfluidic cooling greatly influences the former parameters. Co-design becomes critical in 3D ICs with microfluidic cooling.

Jianyong Xie et al. proposed the electrical-thermal co-simulation of 3D systems with fluidic cooling, joule heating, and air convection effects. The finite-volume formulations of heat equations and the voltage distribution equation for both solid medium and fluid flow are carefully explained. Based on the proposed iterative co-simulation method, package voltage dropped and temperature distribution with fluidic cooling effects can be estimated [22]. Zhimin Wan et al. investigated the co-design of multicore architecture and microfluidic cooling for 3D-stacked ICs, in which a 16-core, ×86 multicore die stacked with a second die hosting an L2 SRAM cache was included. In their works, a multicore ×86 compatible cycle-level microarchitecture simulator was reached and integrated with physical power models. The simulator executed benchmark programs to create power traces that drive thermal analysis. After using a compact thermal model, thermal characteristics under liquid cooling were investigated, and four alternative packaging organizations were compared. With a given pumping power, the greatest overall temperature reduction was reached, with two pin-fin-enhanced microgaps and two tiers, where the high power dissipation tier is on the top. At last, the pin-fin parameters were optimized such that significant improvements in energy instruction were made, and leakage power, as well as the height, the diameter, and the longitudinal and transversal spacing, significantly decreased [23]. Bing Shi et al. studied a hybrid 3D-IC cooling method that combined thermal TSVs with micro-channel liquid cooling structures. The thermal TSVs acted as heat removal agents and as beneficial heat conduction paths to the micro-channel structures. The experimental results demonstrated that the proposed hybrid cooling method provided more cooling capability than thermal TSVs alone but, compared with pure microchannel cooling, required 56% less cooling power [24]. Caleb Serafy presented a scheme for multi-domain co-optimization and co-simulation of 3D central processing unit (CPU) architectures with both microfluidic and air cooling solutions, and demonstrated a paradigm for design space modeling and exploration in the co-design scheme, and discussed possible avenues for improvement of this work in the future [25]. Yue Zhang et al. investigated the electrical and thermal co-design of an interlayer microfluidic heat sink and its experimental test-bed, and analyzed design tradeoffs between heat removal capability and the associated TSV parasitics [26].

## 4. Influence of Microfluidic Cooling on Through Silicon Vias

The thickness of chips or of gaps between chips has been modified to embed microfluidic cooling structures into 3D stacks. The coolant and structure will result in a change in electrical characterization including high frequency. The impact of microfluidic cooling on the electrical performance of TSVs needs to be investigated for 3D ICs. The impact of microfluidic cooling on the electrical characteristics of ICs has been studied. Experimental results have demonstrated a 66.2% leakage current decrease in a complementary metal-oxide-semiconductor (CMOS) chip with microfluidic cooling because of the high cooling capability [27]. Results have also shown the frequency-dependent electrical characteristics of liquid coolants when microfluidic cooling is integrated with an L-band filter to demonstrate a tunable corner frequency of the filter considering the dielectric constant of the coolant [28]. To achieve broadband frequency tunability using distilled water and using methanol–water as a coolant, a tunable RF sensor with a conductor-backed coplanar waveguide was integrated [29]. The coolant between ground and signal interconnects bring about signal propagation, signal integrity, loss, and crosstalk [30].

**Figure 6:**
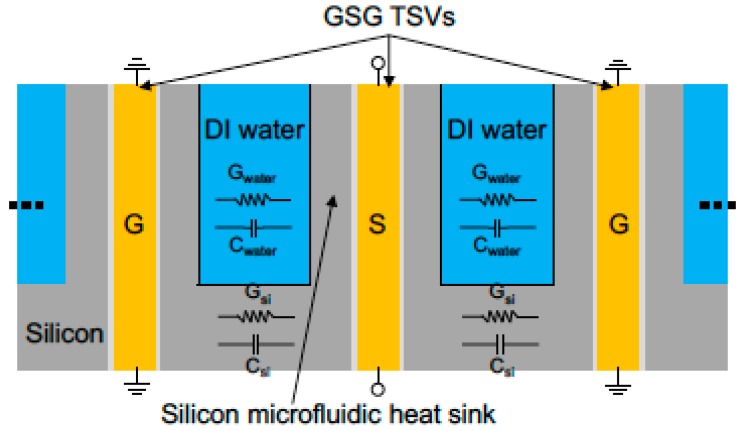
Ground–signal–ground through silicon vias (TSVs) in a microfluidic heat sink. Reproduced with permission from [32].

Hanju Oh et al. sought to determine what is missing for microfluidic cooling to influence TSVs. Their works investigated impacts on insertion loss, TSV capacitance, and conductance of microfluidic cooling, considered the design of a microfluidic cooling testbed containing TSVs using two kinds of heat sinks, and reported a high frequency of microfluidic heat-sink-embedded TSVs in deionized water [31]. The signal propagation in TSVs with integrated microfluidic cooling can be seen in Figure 6, which illustrates the ground–signal–ground TSVs with integrated microfluidic cooling. In such a cooling system, a signal propagating through TSVs will partially encounter capacitance and conductance through silicon and de-ionized (DI) water. Unlike the permittivity of silicon, the permittivity of DI water varied significantly with frequency [32].

**Figure 7:**
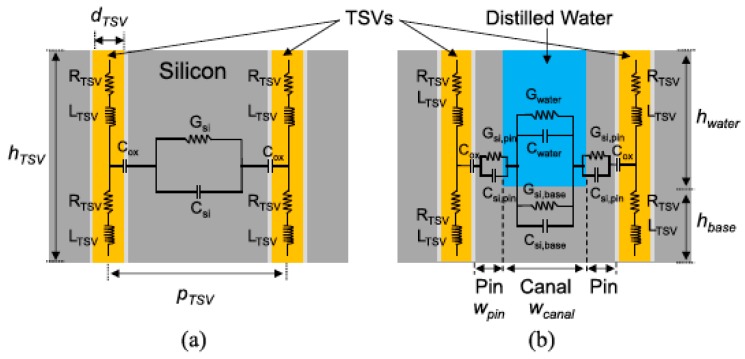
Equivalent circuit models for (**a**) conventional TSVs and (**b**) TSVs with distilled water. Reproduced with permission from [34].

Additionally, Hanju Oh et al. focused on the fabrication of fully isolated TSVs with an aspect ratio of 23:1, investigated the integration of high-aspect-ratio TSVs within a microfluidic heat sink using various fabrication processes, proposed a 3D system with TSVs embedded within interlayer-microfluidic cooling, and described the fabrication and the electrical characterization of high-aspect-ratio TSVs within a micropin-fin heat sink in detail [33]. The distilled water, common acting as a coolant, brought about an impact on the electrical performance of TSVs. Equivalent circuit models of TSVs in a silicon substrate and TSVs in a micropin-fin heat sink filled with distilled water are shown in Figure 7 [34]. This model can be used to analyze the influence of liquid cooling on the electrical performance of TSVs using a microfluidic cooling testbed containing TSVs.

Hanhua Qian et al. presented an accurate steady-state thermal simulator for both sink-cooled and microfluidic cooled 3D ICs. The thermal effect of TSVs at fine granularity was determined by calculating the anisotropic equivalent thermal conductance of a solid grid cell when TSVs are inserted. This model can estimate the thermal effect of TSVs on computationally efficiency and fine granularity. It also considers the entrance influence of microchannels based on the most appropriate thermodynamics for microfluidic cooling [35].

## 5. Microfluidic Cooling Application in Specific Application

Microfluidic cooling was demonstrated to achieve excellent thermal management in 3D-stack-integrated structures. Some projects have focused on 3D stack applications with microfluidic cooling. Caleb Serafy et al. used microfluidic cooling architectures in 3D processor stacks, and explored the new design scheme that was available to computer architects when microfluidic cooling was applied to the 3D chips. With microfluidic cooling, these performance improvements became realizable, and new high performance architectures were achieved when cooling was designed considering the architecture. Results showed a 2.4× increase in average performance when comparing a 3D-stacked memory processor with and without micro-fluidic cooling [36]. The relationship between the performance of a 3D field programmable gate array (FPGA) and micro-channel heat sink design is very complicated and requires a comprehensive method that identifies the optimal design of 3D FPGAs subject to thermo-electrical constraints. Zhiyuan Yang et al. proposed an analysis scheme for 3D FPGAs in which microchannels based on fluidic cooling were embedded to study the influence of channel density on performance and cooling. They provided guidelines for designing 3D FPGAs with micro-channel cooling and identified the optimal design for each benchmark. Optimal design using the scheme can increase the energy efficiency and operating frequency by up to 80.3% and 124.0%, when compared to original 3D FPGA designs with fixed thermal heat sinks [37]. To overcome the limit of microfluidic cooling experiments to silicon with effective heaters representing the heat generating circuitry, a micropin-fin heat sink was etched into the backside of an Altera FPGA with a 28 nm CMOS process. The FPGA was cooled with inlet water temperatures as high as 50 °C, enabling high efficiency through heat exchange directly to ambient air, or waste heat reuse [38].

**Figure 8:**
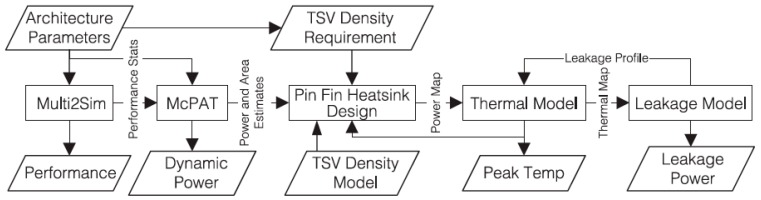
Flowchart of the analysis approach.

Three-dimensional CPUs have also been used to study microfluidic cooling. Caleb Serafy et al. performed a design of microfluidic cooling on a 3D CPU, in which microfluidic cooling was shown as a necessary aggressive cooling solution to unlock the true potential of 3D ICs. After simulating a spectrum of 3D CPU architectures, a 2.3× improvement in performance was reached when microfluidic cooling and floor plan co-optimization was applied [39]. A simulation flowchart for the thermoelectric co-design of 3D CPUs with embedded microfluidic cooling pin-fin heat sinks, which identifies optimal architectural and heat sink design points, is shown in Figure 8, showing the design methodology of a 3D CPU architecture with the microfluidic heat sink that is required to find optimal design choices subject to both physical constraints [40]. Yue Zhang et al., with respect to tier microfluidic cooling, reported on a processor stack in which TSVs with a 23:1 aspect ratio were integrated into a microfluidic heat sink and a vacuum cavity was integrated in the low power tier. Therefore the tier was protected from temperature variation and non-uniformity [41].

High-performance computing is another application with respect to microfluidic cooling. Li Zheng et al. proposed a silicon interposer platform using microfluidic cooling for high-performance 3D computing systems, in which a logic stack was embedded into the microfluidic heat sink in each tier and a memory stack was assembled side by side on a silicon interposer. High-bandwidth signaling between the two stacks was achieved in the system [42]. The thermal experimental results based on the measured thermal resistance showed a 40.1% reduction in the silicon interposer temperature with microfluidic cooling compared to air cooling [43]. Mark D. Schultz et al. published works on the integration of microfluidic cooling with a functional high-performance server. Two-phase flow boiling was proposed as a potential method for cooling high-performance computer systems, and detailed descriptions on the design, fabrication, testing, and characterization of the microfluidic two-phase cooling of a high power microprocessor were given [44,45].

Besides these applications, systems on chips (SoCs) or compound semiconductor devices have been applied. Mohamed M. Sabry et al. demonstrated the potential of power delivery and cooling issues, caused by limitations in Dennard scaling, using a disruptive approach for integrated power generation and cooling based on multiprocessor SoCs. Indeed, this new approach used the coolant fluid as a means of delivering energy to the chips [46]. Wen Yueh et al. presented the design, experimental characterization, and feasibility analysis of integrated in-package fluidic cooling for mobile SoCs. A pin-fin interposer for fluidic cooling was designed and integrated with a commercial SoC. The demonstrated system was integrated with an active low-power piezoelectric pump controlled by the SoC itself and a metal/acrylic-based board-scale heat spreader and exchanger. Different software-based policies in the SoC for controlling the fluid flow based on the SoC’s temperature and performance were implemented and compared. The measurement results demonstrated that in-package fluidic cooling, compared to external passive cooling, improves the SoC’s energy efficiency and reduces the design footprint [47]. To reduce the temperature of high-power X band gallium nitride devices and amplifiers, an integrated microfluidic cooling scheme on multilayer organic liquid crystal polymer substrate has been set up. Experimental results demonstrated the need for dynamic microfluidics in packages to mitigate thermal effects [48,49], and the microfluidic cooling technique improved a GaN monolithic microwave IC amplifier’s gain by over 4 dB, its maximum output power by over 8 dB, and its power efficiency by 3% to 5% [50].

## 6. Thermal Models, Characteristics, and Transmissions in Microfluidic Cooling

One of the important issues to achieve accuracy and efficiency in the thermal management of 3D ICs is to construct thermal models including all relevant parameters. Outmane Lemtiri Chlieh et al. set up a thermal model to predict the overall thermal resistance of the organic heat sink in the case of a moving coolant inside a microfluidic channel based on existing thermal models in the literature, and then fabricated and tested four sets of microfluidic channels with different thicknesses. The temperature measurements of the resistors with different power ratings and sizes agreed with the model predictions [51]. Model simulators will thus be necessary. Wan Z. et al. studied a coupled power-thermal simulator that was used to analyze the influence of on-chip micropin-fin cooling design on both the electrical and thermal performance of 3D ICs when working with real applications and producing hotspot maps. The leakage power accounted for 55.8% of the dynamic power consumption of the chip. An ambient heat transfer coefficient had very little influence on the electrical and thermal performance, while the increasing ambient temperature strongly influences the fluid temperature [52].

For thermal management in GaN-based microelectronic devices with microfluidic cooling, Gunjan Agarwal et al. used finite element analysis to achieve numerical thermal models compatible with heterogeneous integration with conventional silicon-based CMOS devices [53]. Considering the influence of structures, Guilian Wang et al. experimentally and numerically investigated the heat transfer and friction characteristics of microfluidic heat sinks with various structures such as rectangular, triangular, and semicircular ribs. The structures were fabricated on the sidewalls of microfluidic channels by a surface micromachining process and used as turbulators to improve the heat transfer rate of the microfluidic heat sink. The results indicated that the utilizing of micro-ribs provided a better heat transfer rate but also increased the pressure drop penalty for microchannels [54]. However, Xuchen Zhang et al. worked on thermal testbeds with embedded micropin-fin heat sinks. They designed and microfabricated two micropin-fin arrays for experiments. The results obtained from the two testbeds were compared and analyzed, and showed that the density of micropin-fins has a significant impact on thermal performance [55].

## 7. Non-Uniform Heating and Hotspots

Hotspots in 3D ICs arise due to the non-uniform utilization of underlying ICs during chip operation. The hotspot areas and the background area of the chip result in excessive temperature gradients across the chip, which adversely affects chip performance and reliability, as large temperature gradients increase thermal stresses. To tackle hotspot cooling, a number of novel techniques have been proposed, and can be summarized into five classes shown in Table 1.

## 8. Conclusions

The discussion above addresses all available publications using microfluidic cooling in 3D ICs. Most research works were published in the last five years and focused on fundamental principles, models, and application examples in microfluidic cooling. Based on all analyses, several future works are suggested:

- Determining how non-uniform heating can be achieved is important for applying microfluidic cooling to 3D ICs, but no exact methods and models are yet available for evaluating hotspots.
- Digital microfluidics is an effective approach used in the cooling processes, but how drive voltage needs to be reduced when the cooling structures are embedded into 3D stacked ICs.
- More systematic achievements are required in manufacturing, testing, and designing methods when using microfluidic cooling in 3D ICs.

## Figures and Tables

**Table 1:** Summary of the literature review for non-uniform heating and hotspots.

Categories	The Methods Used in the Literatures	References
The analysis of non-uniform heating and hotspots	A novel, low profile jet impingement was given within an individual channel suitable for targeting hotspots in a densely packed circuit, at the low Reynolds numbers prevalent in micro-fluidic applications (Re < 500).	A.M. Waddell et al. (2016) [56]
	A micro-fluidic cooling chip with different-sized hotspots was fabricated to investigate the influence of hotspot characteristics on the cooling ability of the embedded micro-channel.	Y.D. Pi et al. (2016) [57]
	A one-dimensional, semi-empirical approach was presented for quick design of a microchannel heat sink for targeted, energy-efficient liquid cooling of hotspots in microprocessors.	C.S. Sharma (2016) [58]
Using digital microfluidics to solve non-uniform heating	A digital microfluidic cooling platform enabled adaptive cooling in IC design.	P.Y. Paik et al. (2008) [5]
	A cooling method based on high-speed electrowetting manipulation of discrete sub-microliter droplets was achieved under voltage control with volume flow rates in excess of 10 mL/min.	V.K. Pamula et al. (2003) [59]
	An alternative cooling technique based on a recently invented “digital microfluidic” platform was reached.	P.Y. Paik et al. (2008) [60]
	An innovative approach to regulate hotspot temperature was demonstrated by creating a hydrophilic spot (H-spot) on the heater that retains a small droplet while the main coolant droplet passes over the hotspot.	G.S. Bindiganavale (2015) [61]
	A novel digital microfluidic liquid cooling system using electrowetting on dielectric developed for demonstrating and studying hotspot cooling towards electronics thermal management was shown.	G.S. Bindiganavale et al. (2014) [62]
	The high accuracy and consistency in volume of coolant nanodrops dispensed from the reservoir, the fast motion of coolant nanodrops to the hotspot to avoid dry-out, and the simultaneous achievement of both small volume and high frequency of nanodrop that arrives to the hotspot were analyzed.	J.B. Yaddessalage (2013) [63]
	A single-sided digital microfluidic device that enables not only effective liquid handling on a single-sided surface but also two-phase heat transfer to enhance thermal rejection performance was created.	S.Y. Park et al. (2017) [64]
Changing channel clustering for non-uniform heating	An efficient clustering algorithm was used to guide the division of microchannels into clusters and the allocation of cooling resources to each cluster in order to achieve an effective microfluidic cooling with a minimal total flow rate.	H.H. Qian et al. (2011) [65]
	A novel liquid-cooling concept was studied, for targeted, energy-efficient cooling of hotspots through passively optimized microchannel structures etched into the backside of a chip.	C.S. Sharma (2014) [66]
	The model was presented for independent interlayer microfluidic cooling for heterogeneous 3D IC applications.	Y. Zhang (2013) [20]
Using novel structures for non-uniform heating	A single microfluidic loop was demonstrated for the combined and efficient cooling of hotspot and moderate power areas.	D. Lorenzini et al. (2016) [67]
	Non-uniform micropin-fin heat sinks for the cooling of ICs with non-uniform maps were studied.	T.E. Sarvey et al. (2017) [68]
	Fine pitch electrical microbumps and annular shaped fluidic microbumps were achieved to enable high bandwidth die-to-die signaling, embedded microfluidic cooling and power delivery for silicon interposer and 3D integrated electronics systems	L. Zheng et al. (2014) [69]
	Temperature-regulated microvalves were designed for energy-efficient fluidic cooling of microelectronic systems.	H. Azarkish et al. (2017) [70]
	A liquid cooling device was achieved based on a matrix of microfluidic cells with individually flow rate controlling microvalves for temperature uniformities.	G. Laguna et al. (2017) [71]
The test and protocols for non-uniform heating solving methods	Novel thermal testbeds with embedded micropin-fin heat sinks for 3D ICs were created.	X.C. Zhang et al. (2016) [72]
	Microfluidic system protocols for integrated on-chip communication and cooling were demonstrated.	S.A. Wirdatmadja et al. (2017) [73]
